# Deciphering the cellular landscape and genetic underpinnings of fiber diameter determined by dermal papilla cells in fine-wool sheep

**DOI:** 10.3389/fcell.2026.1764812

**Published:** 2026-06-01

**Authors:** Tong Xiao, Tingting Guo, Chao Yuan, Wentao Zhang, Yu Luo, Jianxiang Tang, Zengkui Lu, Bin Liang, Jianbin Liu

**Affiliations:** 1 Lanzhou Institute of Husbandry and Pharmaceutical Sciences, Chinese Academy of Agricultural Sciences, Lanzhou, China; 2 Sheep Breeding Engineering Technology Research Center, Chinese Academy of Agricultural Sciences, Lanzhou, China; 3 Key Laboratory of Animal Genetics and Breeding on Tibetan Plateau, Ministry of Agriculture and Rural Affairs, Lanzhou, China; 4 Gansu Provincial Sheep Breeding Technology Extension Station, Huang ChengTownSunan, China

**Keywords:** dermal papilla cell, fiber diameter, fine-wool sheep, hair follicle, single-cell transcriptome

## Abstract

Wool fiber diameter (FD) is a key economic trait in fine-wool sheep, and its growth process is closely associated with the development and cyclical growth of hair follicles (HFs). Therefore, a comprehensive understanding of the molecular mechanisms regulating HF development is of significant importance. In this study, single-cell RNA sequencing (scRNA-seq) was performed on skin tissues from six half-sibling fine-wool sheep reared under identical conditions but exhibiting different fiber diameters, successfully constructing a transcriptional atlas of the HF microenvironment. A total of 59,732 high-quality cells were captured and systematically annotated into 14 distinct cell types using known marker genes. Each cell type exhibited unique gene expression profiles. Analysis of cell proportions across the three experimental groups revealed that the ultra-fine group had a higher proportion of dermal papilla cells (DPCs), hair follicle stem cells (HFSC), and outer root sheath cells (ORS) compared to the other two groups. Cell-cell communication analysis identified multiple ligand-receptor pairs involved in HF regulation, suggesting that DPCs may play an important role in modulating HF growth and development. Pseudotime trajectory analysis reconstructed the developmental dynamics of the epidermal lineage. Furthermore, we identified that DPCs may exist in three distinct states to perform their physiological functions. To elucidate the molecular mechanisms underlying FD, we identified and analyzed differentially expressed genes across cell types in the ultra-fine, medium-fine, and fine wool groups, and performed cellular-level validation of *CRABP1*, which was significantly upregulated in DPCs, revealing that overexpression of this gene significantly upregulated *PCNA* and *CTNNB1*, suppressed the expression of *SFRP2* and *BMP2*, and subsequently promoted the proliferation of DPCs. This study successfully constructed a single-cell atlas of skin tissues from one-year-old fine-wool sheep, revealing the potential important role of DPCs in HF development and uncovering potential molecular mechanisms of FD. These findings provide new insights into the biology of HF development in sheep and offer valuable references for molecular-targeted precision breeding of ultra-fine wool sheep.

## 1 Introduction

Wool quality primarily depends on the HFs, a vital accessory organ of the skin, whose molecular regulatory mechanisms exhibit high conservation and complexity (Chu et al., 2025). As the production factory for wool, HFs development begins during the embryonic stage. The HFs cycle dynamically cycles through anagen (growth phase), catagen (regression phase), and telogen (resting phase) after birth. Serving as the body’s stem cell reservoir and hair shaft (HS) factory, HFs can participate in remodeling the skin microenvironment by regulating local nerve distribution and vascular networks. Additionally, HFs are involved in multiple physiological functions, such as physical protection, thermoregulation, and sensory perception (Ji et al., 2021), its growth and developmental state directly influences wool traits, including wool FD, length, curvature, et al. Therefore, elucidating the molecular mechanisms driving HFs development is of paramount importance.

FD is one of the key economic traits of wool and a crucial indicator of wool quality. It determines the softness and comfort of wool products. Consequently, as living standards rise, demands for wool product quality have increased, elevating the focus on breeding ultra-fine wool sheep to new heights (Zhang et al., 2023). FD is closely linked to the biological characteristics of HFs, with its underlying mechanisms involving multiple dimensions such as gene regulation, protein expression, and tissue metabolism. Current research on animal fiber diameter primarily focuses on identifying key genes and single nucleotide polymorphisms (SNPs) associated with fiber diameter using genome-wide association studies and candidate gene polymorphisms. RNA-seq analysis on skin samples from coarse-wooled and fine-wooled Angora rabbits to screen for genes potentially influencing rabbit hair FD, such as *LEF1*, *FZD3*, *SMAD3*, and *ITGB6* (Huang et al., 2023). Whole-genome resequencing of eight sheep breeds revealed that genes including *PLCB2*, *PIK3CA*, *GNAI3*, and *GRIA1* may directly or indirectly regulating wool traits, curvature, adipocytes, and androgens to influence FD, thereby promoting the production of finer wool fibers (Zhang et al., 2023). By comparing the gene expression patterns in the skin transcriptomes of Subo Merino (FD: 17.0∼19.0 μm) and Chinese Merino (FD: 19.6∼25.0 μm), 16 differentially expressed genes (DEGs) (*CACNA1S*, *GP5*, *LOC101102392*, *HSF5*, *SLITRK2*, *LOC101104661*, *CREB3L4*, *COL1A1*, etc.) potentially associated with FD were identified were identified (Ma et al., 2023); SNPs genotyping in the *SLIT3* and *ZNF280B* genes of Alpine Merino sheep and subsequent association analysis with FD revealed that *SLIT3* g.478807 C>G and *ZNF280B* g.677 G>A exhibited finer FD, with significant differences compared to other genotypes (Yu et al., 2023).

Non-protein factors (such as vitamin A and vitamin D) regulate skin HF morphogenesis and regeneration. Among these, the active metabolites of vitamin A are isomers of retinoic acid (RA), including cis-retinoic acid and all-trans-retinoic acid (ATRA). Normal RA signaling is crucial for HF morphogenesis; expression of a dominant-negative retinoic acid receptor (RAR) in the epidermis leads to impaired epidermal differentiation and failure to form hair (Charlotte and Watt, 2008). ATRA induces the expression of TGF-β2 in keratinocytes and fibroblasts, thereby promoting the transition of HFs from anagen to catagen (Kerstin et al., 2005). High doses of ATRA inhibit the growth of goat DPCs, resulting in defective HF development and premature entry into the catagen phase (Ma et al., 2018). In addition to the RA signaling pathway, other ligand-receptor systems also play critical regulatory roles in HF morphogenesis and regeneration. Earlier studies have shown that dermally expressed BMP4/7 can form an activation-inhibition feedback loop with the epidermal Edar receptor, ensuring proper spacing between HFs and ultimately leading to the formation of evenly spaced, regularly distributed HF arrays on the skin surface (Chunyan et al., 2006). FGF7, as an important paracrine factor of DPCs, mediates the Wnt signaling pathway to regulate the hair cycle and also enhances FGFR2 expression to promote cell proliferation (Wang et al., 2025b). Furthermore, Wnt signaling can induce dermal BMP4 expression, which in turn promotes the activation of FGF7/FGF10 signals in the dermis, ultimately providing feedback to regulate the proliferation of epidermal basal cells (Xiao-Jing et al., 2014). The crosstalk among these signaling pathways constitutes the core framework of the regulatory network governing hair follicle development.

The ultra-high-resolution cell resolution capability of scRNA-seq provides robust technical support for investigating HFs heterogeneity and the complex biological processes regulating FD (Chovatiya et al., 2021). Currently, scRNA-seq has been maturely applied to HFs biology studies across multiple sheep breeds, such as Hu sheep (Shanhe et al., 2021), Ordos fine-wool sheep (Li et al., 2024), cashmere goats (Ge et al., 2022; Feng et al., 2022), Dazu Black Goat (Zhang et al., 2025), among others. However, the molecular genetic mechanisms underlying the variation in FD in fine-wool sheep is rarely reported. Compared with coarse wool, fine wool is softer, more uniform, and has higher textile value. In contrast to cashmere, fine wool offers greater yield, longer fiber length, and is not subject to seasonal fluctuations. Therefore, this study systematically analyzed the heterogeneity of HFs in Alpine sheep skin, using scRNA-seq. It further investigated the DEGs in fine-wool group (FD: 20.1∼21.5 μm), medium-fine wool group (FD: 18.1∼20.0 μm), and ultra-fine wool group (FD: <18 μm) Alpine Merino sheep. This analysis revealed potential molecular mechanisms influencing HFs development, which in turn affect wool FD traits, aiming to provide references for new ultra-fine wool sheep breeding.

## 2 Materials and methods

### 2.1 Experimental animals

The experimental animals were obtained from the fine-wool sheep core breeding farm (Gansu Provincial Sheep Breeding Technology Promotion Station, Sunan, Gansu, China). Under standardized and identical housing conditions, six yearling Alpine Merino ewes from the same paternal lineage were selected as experimental subjects. Based on average FD, they were assigned to three groups: an ultra-fine group (n = 2, FD: <18.0 μm), a medium-fine group (n = 2, FD: 18.1∼20.0 μm), and a fine group (n = 2, FD: 20.1∼21.5 μm). Detailed FD data are provided in Supplementary Table S4. Using a skin biopsy punches (Acu-Punch® Disposable Skin Biopsy Punches 10 mm, Florida, United States), three pieces of skin tissue were collected from a site located approximately one palm’s width posterior to the scapula. One sample was immediately placed in a specialized tissue preservative for subsequent scRNA-seq. The second sample was fixed in 4% paraformaldehyde solution for histological preparation and sectioning. The third sample was snap-frozen in liquid nitrogen for potential future analysis. All animal procedures were conducted in accordance with a protocol approved by the Animal Ethics Committee of the Institute of Animal Science, Chinese Academy of Agricultural Sciences (Approval No: SYXK-2014-0002; Approval Date: 13 May 2022).

### 2.2 Preparation of single-cell suspensions and cDNA library construction

The obtained skin tissue was rinsed with PBS to remove blood and residual subcutaneous fat. It was then digested at 37 °C for 30 min using a mixed enzyme solution containing 2.5 mg/mL neutral protease and 2.5 mg/mL trypsin. Digestion was terminated by adding 10% fetal bovine serum. The resulting cell suspension was filtered through a 40 μm cell strainer. After centrifugation, the cell pellet was resuspended in PBS, followed by the addition of 1 mL red blood cell lysis buffer to remove residual erythrocytes. Dead cells were removed using a Dead Cell Removal Kit (Miltenyi Biotec, Germany). The pellet was resuspended again, and cell viability and live cell count were assessed using Trypan blue staining on a Countess II automated cell counter. The final cell concentration was approximately 1,100 cells/μL, with viability exceeding 94%.

Following the manufacturer’s instructions for the Chromium Single Cell 3′v3 Reagent Kit (10x Genomics, Pleasanton, CA, United States), oil droplet-encapsulated single-cell Gel Beads-in-Emulsion (GEMs) were generated. Within the GEMs, cells were lysed to release mRNA, which was then captured by barcoded oligonucleotides on the Gel Beads. Reverse transcription was performed to synthesize cDNA. The cDNA concentration was quantified using Qubit assays. Qualified cDNA was used directly to construct 3′ gene expression libraries. Finally, the constructed libraries were subjected to high-throughput sequencing on an Illumina platform using a paired-end sequencing mode.

### 2.3 ScRNA-seq data processing and analysis

The raw sequencing data were processed and analyzed with the 10× Genomics Cellranger analysis pipeline. Using the STAR aligner, the reads were aligned to the reference genome (https://www.ncbi.nlm.nih.gov/datasets/genome/GCF_000298735.2/) for cell barcode and UMI (Unique Molecular Identifier) counting, thereby generating a cell-by-gene expression matrix. After quality control, the Seurat package was used for data normalization, dimensionality reduction, cluster identification, and DEGs analysis. In accordance with the standard workflow, Monocle was then applied to reconstruct pseudo-temporal cell differentiation trajectories. Finally, cellchat analysis was performed to map ligand-receptor interaction networks among cell clusters.

### 2.4 Isolation, purification, and identification of DPCs and histological structure observation

The isolation, purification, and identification of DPCs were performed according to the established protocol of our research group (Haig et al., 2014). For histological analysis, fixed tissue samples were trimmed, dehydrated through a graded ethanol series, cleared in xylene, and embedded in paraffin. Sections were prepared after the paraffin had solidified. The paraffin sections were dewaxed and rehydrated. They were then stained with hematoxylin for 3∼5 min, differentiated, blued, dehydrated through an ethanol series, and counterstained with eosin for 2 min. Finally, the sections were dehydrated again, cleared in xylene, and mounted with neutral resin for microscopic examination and imaging. For immunohistochemistry, paraffin sections were dewaxed and rehydrated, followed by antigen retrieval. After cooling naturally, the sections were washed three times. Endogenous peroxidase activity was quenched by incubation with 3% hydrogen peroxide. The sections were then blocked with 3% bovine serum albumin (Sigma-Aldrich, Livonia, Michigan, United States) at room temperature for 30 min. After removing the blocking solution, the primary antibody was applied and incubated overnight at 4 °C. Subsequently, the corresponding secondary antibody was added and incubated at room temperature for 50 min. Positive signals were visualized using diaminobenzidine substrate. The reaction was stopped by rinsing with tap water, followed by counterstaining with hematoxylin for approximately 3 min. After dehydration, clearing, and mounting with neutral resin, the sections were examined and photographed under a microscope.

### 2.5 Construction of overexpression and interference vectors and cell transfection

Primers for amplifying the target gene coding sequence (CDS) were designed according to the reference sequence obtained from the NCBI database. The amplified fragment was generated by double digestion with Hind *III* and EcoR *I* and then directionally cloned into a eukaryotic expression vector, resulting in the recombinant plasmid pcDNA3.1(+). The negative control vector, the recombinant plasmid, and siRNA were designed and synthesized by GenePharma Co., Ltd. (Suzhou, China). Relevant sequence information is provided in Supplementary Table S1. Cultured DPCs up to the fifth generation were plated. When confluence reached 60%∼70%, the overexpression vector and interference vector were transfected into DPCs following the protocol provided by the transfection kit (Zeta Life, United States). Twenty-four hours post-transfection, the cells were harvested for subsequent experiments.

### 2.6 Real-time fluorescent quantitative PCR (RT-qPCR)

Total RNA was extracted from DPCs using the Trizol method, and determined its concentration. The extracted RNA was reverse transcribed into cDNA using the Prime Script™ RT Reagent Kit with gDNA Eraser (Vazyme, Nanjing, China). RT-qPCR primers were designed against the target gene coding sequences retrieved from the NCBI database, using the Primer-Blast tool. All primers were synthesized by Sangon Biotech Co. Ltd (Shanghai, China) (sequences listed in Supplementary Table S2). Reaction mixtures were prepared in RNase-free tubes, with β-actin used as the internal reference gene. The thermal cycling protocol was as follows: initial denaturation at 94 °C for 30 s; followed by 40 cycles of denaturation at 94 °C for 10 s and annealing/extension at 60 °C for 30 s.

### 2.7 Cell proliferation assay

Cell proliferation assay was used EDU proliferation assay and CCK-8 assay.

EDU Assay: Cell proliferation was detected using an EDU assay kit (Biyuntian, China). At 24 h post-transfection, cells were incubated with EDU working solution (diluted 1:500 in culture medium) for 2 h at 37 °C. After discarding the medium, cells were fixed with 4% paraformaldehyde for 15 min at room temperature, followed by three washes with PBS containing 3% BSA. Permeabilization was performed with 0.3% Triton X-100 for 15 min. After washing, 500 μL of Click reaction solution was added per well and incubated in the dark for 30 min. Following another wash, nuclei were stained with Hoechst 33,342 for 10 min. Images were acquired using a fluorescence microscope.

CCK-8 Assay: Transfected DPCs were seeded into 96-well plates. At the indicated time points (2, 12, 24, 36, and 48 h), CCK-8 solution was added in the dark and incubated at 37 °C under 5% CO_2_ for 2 h. Absorbance at 450 nm was measured using a microplate reader (IMPLEN, Palo Alto, CA, United States).

### 2.8 Immunohistochemistry

Sections were dewaxed and rehydrated to water. Antigen retrieval was performed using citric acid antigen retrieval solution. After natural cooling, the slides were placed in PBS and washed three times on a decolorizing shaker for 5 min each wash. The sections were then immersed in 3% hydrogen peroxide solution and incubated for 25 min at room temperature in the dark to block endogenous peroxidase activity. Within the immunohistochemistry circle, 3% BSA was added to evenly cover the tissue, followed by blocking for 30 min at room temperature. The blocking solution was gently flicked off, and the primary antibody was applied and incubated overnight at 4 °C. After washing, the secondary antibody was added and incubated for 50 min at room temperature. Following another wash, DAB chromogen solution was added when the sections were slightly dry, and the color development time was controlled under a microscope. The reaction was terminated by rinsing with tap water. The sections were then counterstained with hematoxylin, dehydrated, cleared, and mounted with neutral resin. Results were observed and interpreted under a light microscope. Antibody information is presented in Supplementary Table S3.

### 2.9 Data statistics and analysis

Statistical analysis was performed using SPSS 22.0 software (IBM, Armonk, United States). For RT-qPCR data, relative quantification was calculated using the 2^−ΔΔCT^ method, and comparisons were evaluated by one-way ANOVA. Each experiment included at least three biological replicates. Data are presented as the “mean ± standard deviation (mean ± SD).” Statistical significance is denoted as follows: *P < 0.05, **P < 0.01, and ***P < 0.001. All bar graphs were generated with GraphPad Prism 10.0.

## 3 Results

### 3.1 ScRNA-seq for cell type identification in fine-wool sheep skin tissue

**FIGURE 1:**
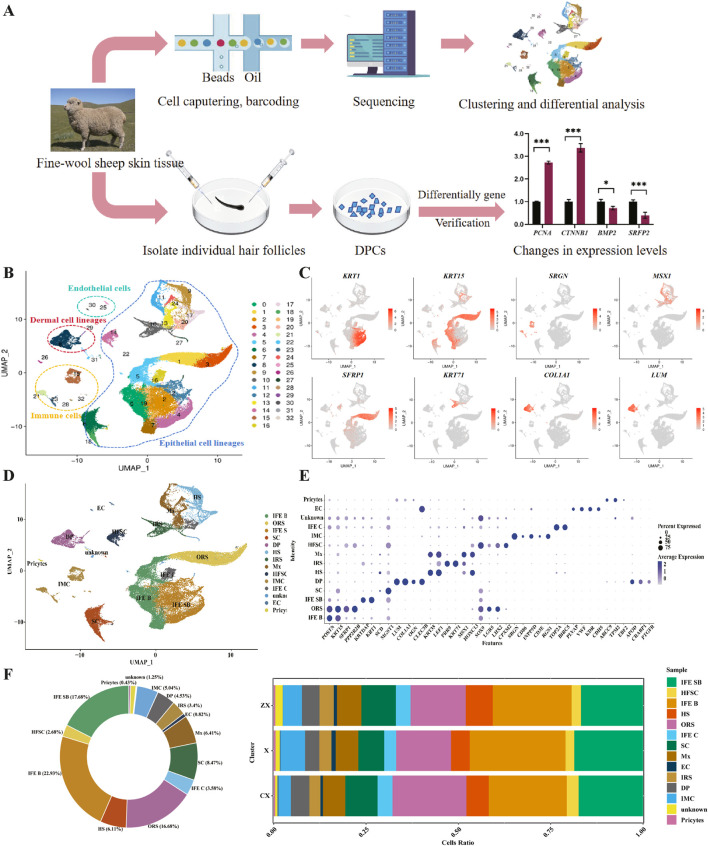
ScRNA-seq reveals annotated cell types in fine-wool sheep skin tissue. **(A)** Experimental workflow overview. **(B)** UMAP plot showing characteristic cell types in skin tissue. **(C)** UMAP visualization of marker genes for major cell types (e.g., epidermis, dermis, immune cells). **(D)** Cluster-specific annotations on the UMAP plot. **(E)** Bubble plot displaying expression of marker genes used for cell-type annotation. **(F)** Proportions of cells across different cell types.

To deeply analyze cellular heterogeneity during HF development and elucidate the molecular mechanisms underlying FD variation of fine-wool sheep, we performed scRNA-seq on skin tissue from one-year-old fine-wool sheep with ultra-fine, medium-fine, and fine-wool FD, all sampled individuals were paternal half-siblings (Figure 1A). Across the six samples representing three FD groups, an average of ≥18,764 cells were captured per sample, with at least 38,360 genes detected and a cross-sample genome alignment rate >96%. After quality control, high-quality cells were retained for downstream analysis. In total, scRNA-seq data from 55,048 cells were analyzed, comprising 17,401 cells from the ultra-fine wool group, 19,865 cells from the medium-fine wool group, and 17,782 cells from the fine wool group (Supplementary Table S5).

Following UMAP dimensionality reduction and clustering of all cells from the three FD groups, we annotated each cell cluster by integrating its uniquely expressed genes (Supplementary Tables S6, S7) with known classical HF cell marker genes. Our work distinguishes 14 distinct cell types within the cluster, which were categorized into the epithelial cell lineage (clusters 0, 1, 2, 3, 4, 5, 6, 7, 9, 10, 11, 12, 13, 14, 16, 17, 18, 19, 20, 22), dermal cell lineage (clusters 8, 29), endothelial cell lineage (clusters 25, 30), immune cell lineage (clusters 15, 21, 23, 28, 32), and one unknown cell lineage (cluster 31) (Figures 1B,D). Key cell populations and their marker genes included: Interfollicular epidermis basal (IFE B, clusters 0, 5, 7, 19), expressing *KRT15*, *POSTN*, *KRTDAP*, *TOP2A* (Gu and Coulombe, 2007; He et al., 2020), etc. Interfollicular epidermis super-basal (IFE SB, cluster 2, 4, 12), Interfollicular epidermis cycling (IFE C, clusters 16, 20); Dermal cell lineage (clusters 8, 29) highly expressing *LUM*, *COL1A1*, *APOE* (Gupta et al., 2019; Ying et al., 1997); IRS (clusters 10, 27), highly expressing *KRT71*, *GATA3* (Joost et al., 2020); Hair follicle stem cell (HFSC, cluster 14), highly expressing *LGR5*, *SOX9* (Gerhards et al., 2016); Pericyte cell (cluster 26), highly expressing *ABCC9* and *TPM2* (Xuxu, 2023); ORS cell (clusters 1, 3), highly expressing *SFRP1* and *TAGLN* (Gerhards et al., 2016); Matrix (Mx, clusters 11, 13, 24) population highly expressing *MSX1* and *HOXC13* (Feng et al., 2022) (Figures 1C,E).

Furthermore, we calculated the proportions of cells among the various cell types. Overall, epithelial cells constitute the largest proportion across all cell types. When comparing differences between FD groups, we found that the ultra-fine group exhibited higher proportions of DPCs, ORS, and HFSC than the other two groups, while the proportions of IMCs and endothelial cells were lower compared to the other two groups (Figure 1F).

### 3.2 Immunohistochemical validation of marker genes

**FIGURE 2:**
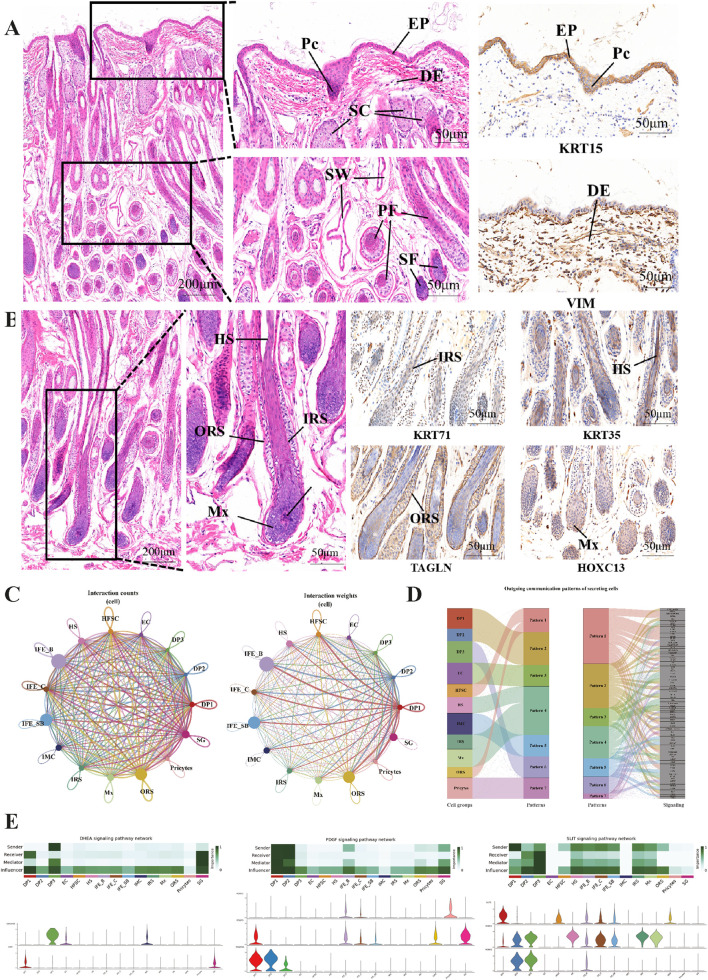
Skin Structure Observation, Immunohistochemistry and Schematic diagram of ligand-receptor communication networks among cell types. **(A)** Histological observation and immunohistochemistry (IHC) of the epidermis and dermis (Note: Staining results are indicated as blue for negative, brown for positive, and dark brown for strong positive.) **(B)** Histological observation and IHC of HF structures. (Abbreviations: Pc: Placode; Ep: epidermis; DE: dermis; SG: sebaceous gland; SW: sweat gland; PF: primary HF; SF: secondary HF; HS: hair shaft; IRS: inner root sheath; ORS: outer root sheath; DP: dermal papilla; Mx: matrix.) **(C)** Number of ligand-receptor interactions (left) and interaction strength (right) among different cell types; **(D)** Visualization of cellular output signal patterns. **(E)** PDGF, DHEA, SLIT signaling pathway network and violin diagram.

Skin samples were collected from the posterior border of the scapula at a width equivalent to the span of one palm. Histological sections were prepared and examined using hematoxylin and eosin (HE) staining. The results showed the skin consists of three layers: epidermis, dermis, and subcutaneous tissue. The epidermis is composed of stratified squamous epithelium, which is arranged sequentially from the inner to the outer layers as the basal layer, spinous cell layer, granular layer, and stratum corneum. The dermis consists of dense connective tissue rich in collagen fibers and contains skin appendages such as HFs, sweat glands (SWs), sebaceous glands (SGs), arrector pili muscles, nerves, and blood vessels. Based on their developmental stage, HFs can be classified into primary follicles (PFs) and secondary follicles (SFs). In fine-wool sheep, SFs are further subdivided into original secondary follicles (OSFs) and secondary-derived follicles (SDFs), the latter of which form through the redifferentiation of OSFs (Ma, 2024). Collagen fibers divide the dermis into multiple follicular units, each comprising 2∼3 PFs and multiple SFs, PFs are typically accompanied by SWs and SGs, whereas SFs have smaller duct diameters and lack SWs (Figure 2A).

The basic structure of HFs is highly conserved, consisting from inner to outer of the hair shaft (HS), inner root sheath (IRS), outer root sheath (ORS), and connective tissue sheath (CTS) (Oshima et al., 2001). The HS occupies the central position within HFs and is composed of highly keratinized epithelial cells. The portion extending beyond the skin surface is termed the hair fiber, while the segment embedded within the skin constitutes the hair root (Zeng et al., 2017). Surrounding the HS is the IRS, a relatively rigid structure composed of keratinized cells that provides support to maintain the shape of the HS (Wang, 2023). The ORS originates from the Malpighian layer of the epidermis and tightly envelops the IRS and HS along their periphery (Paus et al., 1994) (Figure 2B).

Immunohistochemistry was performed on skin tissue sections to validate the marker genes and their corresponding cell types. The results indicated strong positive expression of KRT15 in epidermal cells and of VIM in dermal cells. Positive expression was observed for KRT35 in the HS, KRT71 in the IRS, and HOXC13 in the matrix (Mx), while TAGLN showed strong positive expression in the ORS. These immunohistochemical findings confirm the reliability of the cell type annotations (Figures 2A,B).

### 3.3 Epidermal-dermal interactions jointly regulate HF development

The process of follicular morphogenesis is highly complex, primarily regulated by mesenchymal–epithelial signaling interactions between the epidermis and dermis, which coordinate the proliferation and differentiation of epithelial and dermal cells, ultimately leading to the formation of complete HF structures (Zhang et al., 2023). Therefore, to elucidate the underlying interactions driving cellular heterogeneity and cell-type transitions during HF development, we performed cell–cell communication analysis on all annotated cell types using CellChat. The results revealed extensive and intricate ligand–receptor interactions among all cell types, with epithelial cells playing a dominant role in these interactions. Among them, IFE B and IFE SB were the most prominent, while ORS, SG, and DP also exhibited substantial interaction strengths (Figure 2C). Analysis of potential outgoing signaling across cell types highlighted a high degree of specialization and division of labor in HF signaling output. The DPCs and HFSCs emerged as active signal senders. HFSCs were predominantly enriched in signaling pathways such as FGF and EPHA, whereas DPCs transmitted signals through pathways including SEMA3, RA, and IGFBP (Figure 2D). Furthermore, in the analysis of classic signaling pathways involved in HF development, such as BMP, FGF, IGF, and IGFBP, DPCs consistently served as the primary signal senders (Supplementary Figure S3).

We further analyzed specific ligand–receptor pairs within each pathway. In the BMP signaling pathway, BMP4 and BMP2 were identified as key ligands, mainly expressed in DPCs (Supplementary Figure S4A), and they cooperated with the canonical receptors BMPR1A and BMPR2 to regulate overall BMP pathway activity. In the FGF pathway, FGF10 and FGF7 were the predominant ligands, also highly concentrated in DPCs, while FGFR2 functioned as the core receptor, receiving most ligand inputs and distributed across various cell types (Supplementary Figure S4B). The TGF-β pathway primarily operated through the TGFB1–TGFBR1 and TGFB3–TGFBR1 pairs, with TGFBR1 widely expressed across multiple cell types but most notably in DPCs (Supplementary Figure S4C). Interestingly, we also found that DPCs acted as the main signal senders in regulating the PDGF, DHEA, and SLIT signaling pathways (Figure 2E).

### 3.4 Reconstruction of the epidermal differentiation process

**FIGURE 3:**
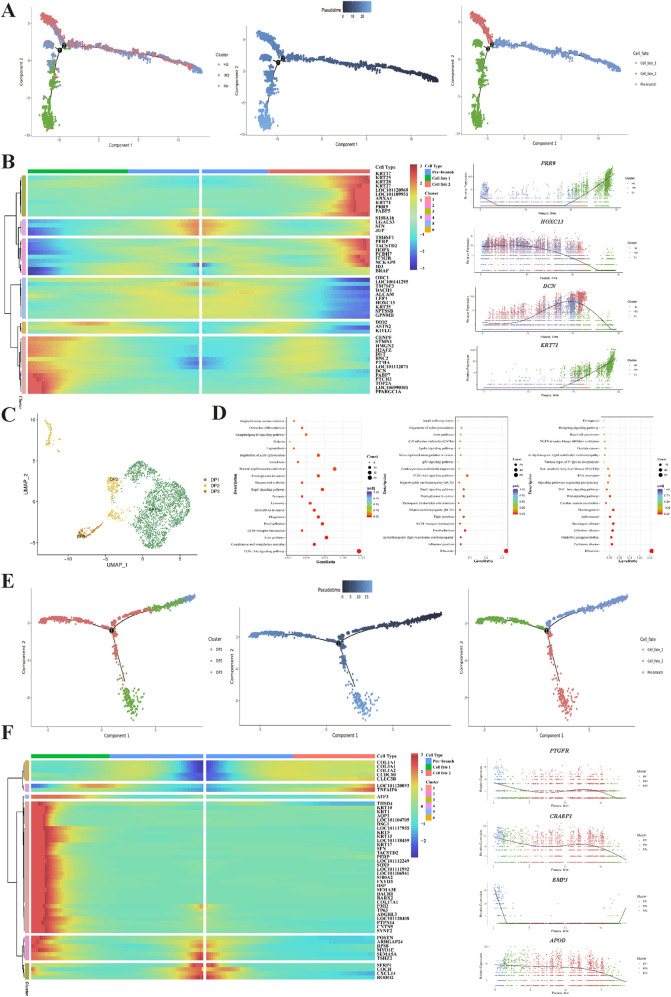
Reconstruction of Epidermal Lineage and DPCs Differentiation Trajectories. **(A)** Differentiation trajectories of Mx, IRS, and HS. **(B)** (Left) Heatmap of differentially expressed genes along the pseudo-time trajectory. (Right) Expression patterns of HOXC13, KRT71, PRR9, and DCN along pseudo-time. **(C)** Three subpopulations were ultimately identified; **(D)** KEGG enrichment analysis of DPCs subpopulations; **(E)** Pseudo-time analysis of three DPCs subpopulations; **(F)** Heatmap of DEGs along pseudo-time trajectories (left), Expression profiles of *APOD*, *CRABP1*, *BMP3*, and *PTGFR* along pseudo-time trajectories (right).

Through pseudo-time analysis, the differentiation trajectory of epidermal cells during HF development was reconstructed. At the onset of the HF growth phase, HFSCs are first activated within the hair germ and subsequently differentiate into Mx. These Mx cells represent an intermediate cell type between HFSCs and their differentiated lineages, continuously giving rise to structures such as the IRS and the companion layer (Zhang and Chen, 2024). Accordingly, this study selected relevant HF epidermal cell clusters—Mxs, IRS, and HS (clusters 9, 10, 11, 13, 17, 24) — to infer lineage developmental trajectories using pseudo-time analysis. As anticipated, the pseudo-time trajectory revealed two distinct branches (Figure 3A), clearly illustrating the sequential differentiation process of cell types. Among these, Mxs were predominantly located in the pre-branch segment, DEGs indicated high expression of genes such as *S100A16*, *SFN*, and *JUN* in this branch. HS mainly clustered in the cell fate 1 branch, with DEGs revealing elevated levels of genes such as *TOP2A*, *DCN*, *FABP7*, and *DUT*. IRS were primarily found in the cell fate 2 branch, where DEGs included *KRT17*, *KRT25*, *KRT71*, and *PRR9* (Figure 3B). Furthermore, the IRS branch specifically upregulates *PRR9* and *KRT71*, whereas *HOXC13* and *DCN* show minimal expression in these cells (Marcin et al., 2013). These findings align closely with the expected differentiation process, confirming the pseudo-temporal trajectory from Mx cells to IRS.

### 3.5 Reconstruction of the of DPCs differentiation process

Based on cell communication analysis revealing that DPCs are one of the crucial cell types influencing HF development, this study further performed unsupervised subclustering of the preliminarily identified DPCs. Clustering analysis identified a total of 8 DPC subpopulations (Supplementary Figure S5A–C), which were ultimately categorized into three distinct subpopulations—DP1, DP2, and DP3—based on correlation heatmaps and specifically highly expressed genes for each cluster (Figure 3C). KEGG enrichment results demonstrated functional divergence among these three subpopulations, highlighting the functional heterogeneity of DPCs. The DP1 subpopulation was enriched in pathways such as the PI3K-Akt signaling pathway, focal adhesion, ECM-receptor interaction, and regulation of actin cytoskeleton, suggesting its primary role in sensing the HF microenvironment and integrating pro-survival signals. The DP2 subpopulation showed enrichment in pathways including cell adhesion molecules, Rap1 signaling pathway, and PI3K-Akt signaling pathway, indicating its predominant functions in cytoskeletal remodeling, intercellular adhesion, and associated regulation of angiogenesis. The DP3 subpopulation was enriched in pathways such as the Hedgehog signaling pathway, Wnt signaling pathway, TGF-β signaling pathway, and Signaling pathways regulating pluripotency of stem cells, implying its important role in modulating HF development (Figure 3D).

Subsequently, pseudotime analysis was performed to reconstruct the developmental trajectory among DPC subpopulations. The results revealed that starting from DP3, two distinct branches emerged: branch 2 mainly composed of DP1 cells and branch 1 consisting of DP2 cells, indicating a continuous process from a primitive state toward divergent fate commitments among DPC subtypes (Figure 3E). A differential gene heatmap illustrated the gene expression changes driving these state transitions. In the pre-branch state, genes such as *POSTN*, *SFRP1*, and *COCH* were highly expressed. Fate 1 state specifically expressed genes including *KRT1*, *KRT10*, and *KRT15*, whereas fate 2 showed high expression of *COL1A1*, *COL1A2*, and *CLEC3B*. Additionally, this study found that the expression of *APOD* changed along the developmental timeline, primarily decreasing along the DP2 branch. Although the expression of *CRABP1* and *PTGFR* generally declined overall, it exhibited fluctuations along the DP1 branch (Figure 3F).

### 3.6 Intergroup differentially expressed genes associated with wool FD

**FIGURE 4:**
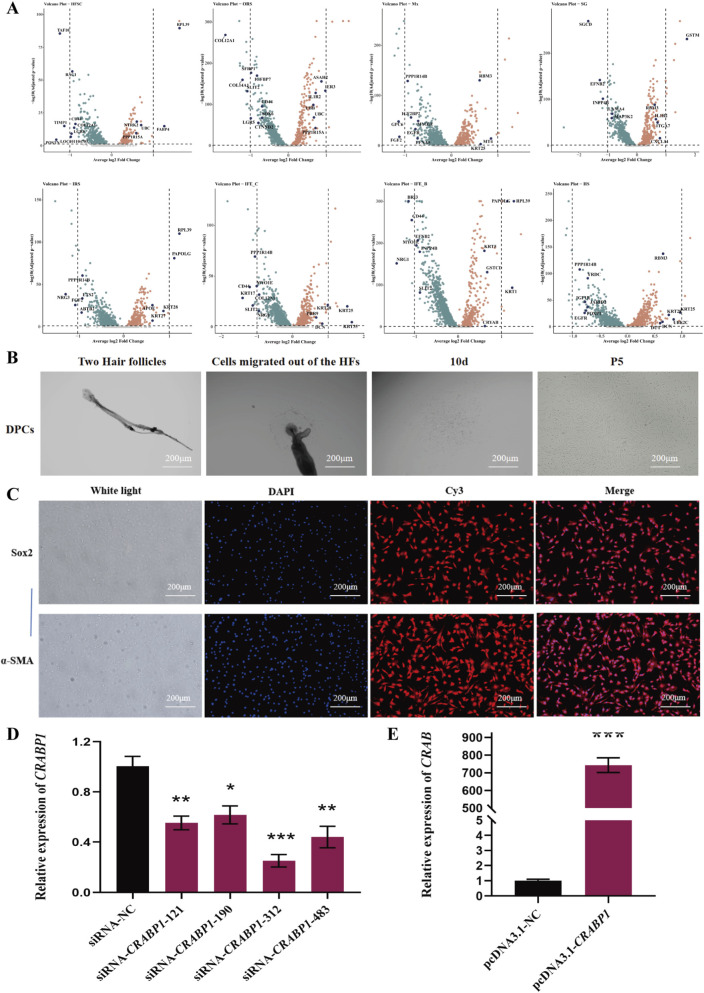
Differential Gene Expression and Its Effects on DPCs. **(A)** Volcano plot of DEGs between ultra-fine and fine wool sheep. **(B)** Completely isolated HF structure (left) and DPCs growth (right). **(C)** Immunofluorescence staining of α-SMA (encoded by *ACTA1*) and SOX2 in isolated DPCs. Cell nuclei appear blue (DAPI), target proteins appear red. **(D)** Expression of *CRABP1* interference fragment in DPCs post-transfection. **(E)** Expression of *CRABP1* overexpression vector in DPCs post-transfection.

FD is closely linked to the biological characteristics of HFs, with underlying mechanisms spanning multiple dimensions such as gene regulation and tissue metabolism. As the foundation of wool growth, the developmental state of HFs directly influences FD. Epithelial-mesenchymal interactions between DPCs and epithelial cells regulate wool coarseness and curvature (Cédric and Elaine, 2009); HFs density and size also influence FD trait (Elliott et al., 1999; Moore et al., 1989). Therefore, to explore the potential molecular mechanisms affecting FD, we employed the Seurat algorithm to compare DEGs across different cell types between the ultra-fine and fine wool groups, and between the medium-fine and fine wool groups (Figure 4A). The comparison of DPCs between the ultra-fine and fine wool groups revealed 147 significantly up- and downregulated genes. Among the significantly upregulated genes, *CRABP1* (Charlotte and Watt, 2008; Sun, 2024), *LGR5* (Jaks et al. 2008; Polkoff et al., 2022), *COL1A1* (Chang et al., 2024; Yue et al., 2024), and *KRT10* (Tina et al., 2023; Huang et al., 2024) have been shown to play critical roles in HF development. We focused on *CRABP1*, as previous studies indicate its specific expression in the DPCs and its function as a retinoic acid-binding protein that regulates hair curvature in mice (Okano et al., 2012). In general, thicker hairs tend to be straighter, while finer hairs often exhibit curvature (Zhang et al., 2023). Therefore, we speculate that this gene plays a crucial role in regulating FD. We also compared DEGs in other cell types—such as HFSCs, Mx, IRS, and ORS—between the ultra-fine and fine wool groups, as these cells may also influence FD (Supplementary Table S8). Additionally, we compared DEGs across cell types between the medium-fine and fine wool groups. In DPCs, 18 genes were significantly up- or downregulated. Notably, the *MYOC* gene was significantly downregulated in both the ultra-fine vs. fine and the medium-fine vs. fine comparisons. This gene has been previously implicated in the regulation of FD (Pu et al., 2024). Together, these findings offer valuable insights for investigating the molecular mechanisms that influence FD.

### 3.7 Effects of *CRABP1* on DPCs proliferation

#### 3.7.1 Isolation, purification, and identification of DPCs

On the sixth day post-isolation, DPCs began to migrate out from the hair bulb region. They initially displayed elongated triangular or polygonal morphologies and subsequently expanded radially outward. As cell density increased due to proliferation, the cells progressively lost their original shapes and formed dense clusters. After reaching stable growth, the cells were purified by differential digestion using 0.25% trypsin, ultimately yielding well-growing, spindle-shaped, irregular DPCs (Figure 4B). To further verify experimental accuracy, the purified DPCs were then identified via immunofluorescence staining using the classic DPC marker proteins α-SMA and SOX2. Results confirmed significant expression of both proteins in the isolated cells (Figure 4C). These findings confirm that the isolated cells are authentic DPCs suitable for subsequent experiments.

#### 3.7.2 Effects of overexpressing *CRABP1* on DPCs proliferation

**FIGURE 5:**
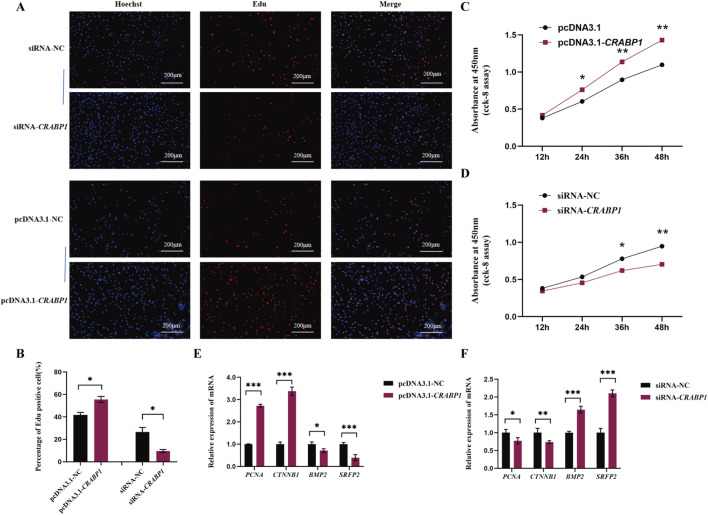
Effects of *CRABP1* on DPCs proliferation. **(A)** Effects of *CRABP1* overexpression and knockdown on DPCs proliferation; red fluorescence represents EDU, blue fluorescence represents Hoechst. **(B)** Statistics of EDU-positive cells after *CRABP1* overexpression and knockdown. **(C)** OD value curves for *CRABP1* overexpression at 12 h, 24 h, 36 h, and 48 h. **(D)** OD value curves for *CRABP1* knockdown at 12 h, 24 h, 36 h, and 48 h. **(E)** Effects of *CRABP1* overexpression on the expression of genes related to HF growth and development. **(F)** Effects of *CRABP1* knockdown on the expression of genes related to HF growth and development.

This study focused on *CRABP1*, one of upregulated DEGs in DPCs of both ultra-fine and fine wool groups. Knockdown of *CRABP1* significantly reduced its mRNA expression levels, with the most effective suppression observed in the siRNA- *CRABP1*-312 (*P* < 0.01, Figure 4D). Conversely, qRT-PCR confirmed a significant upregulation of *CRABP1* in DPCs following its overexpression (*P* < 0.001, Figure 4E). To investigate the functional impact of *CRABP1* overexpression, EDU assay results demonstrated that it significantly promotion cell proliferation (*P* < 0.05, Figures 5A,B), a finding further supported by CCK-8 assay results (Figure 5C). Mechanistically, qPCR analysis revealed that *CRABP1* overexpression robustly upregulated the expression of *PCNA* (*P* < 0.001) and *CTNNB1* (*P* < 0.001), while suppressing the expression of *BMP2* (*P* < 0.05) and *SFRP2* (*P* < 0.001) (Figure 5E).

#### 3.7.3 Effects of *CRABP1* knockdown on DPCs proliferation

To examine the opposing functional effects, *CRABP1* knockdown in DPCs significantly inhibited cell proliferation, as shown by EDU assay results (*P* < 0.05, Figures 5A,B). This suppression of proliferation was similarly confirmed by CCK-8 assay results (Figure 5D). At the molecular level, analysis of mRNA expression revealed that *CRABP1* knockdown significantly downregulated *PCNA* (*P* < 0.05) and *CTNNB1* (*P* < 0.01) while upregulating *BMP2* (*P* < 0.001) and *SFRP2* (*P* < 0.001) expression (Figure 5F).

## 4 Discussion

HFs are important accessory structures on mammalian skin. Current research on HFs primarily focuses on identifying key genes and signaling pathways that regulate their development, with the goal of treating related diseases or improving the quantity and quality of animal fur products. FD is a crucial economic trait of wool, directly influenced by the growth and development of HFs. Therefore, exploring the molecular mechanisms of HF morphogenesis and development is highly significant for the selective breeding of fine-wool sheep breeds. ScRNA-seq technology provides powerful technical support for studying the complex process of HF morphogenesis and development. In recent years, several studies have successfully utilized this technology to analyze the heterogeneity of skin HFs across different species (Chovatiya et al., 2021; Feng et al., 2022; Wang et al., 2024; Qingbo et al., 2023). Nevertheless, the intricate regulatory mechanisms between the epidermis and dermis, as well as the mechanisms governing FD in sheep, still require further elucidation.

In this study, scRNA-seq was employed to obtain 59,732 cells from three distinct FD groups, annotating 14 known HF cell types. This demonstrates the cellular stability underlying the functioning of the skin tissue microstructure. Immunohistochemical validation of several marker genes on skin tissue sections showed consistency with the single-cell analysis results. Additionally, we identified the isolated DPCs *in vitro* using the classical marker gene *SOX2*, which is commonly employed in model organisms such as humans and mice, but it was not detected in our single-cell data. By utilizing CellChat to analyze the intercellular interactions driven by epidermal-dermal crosstalk, we found that DPCs may establish close connections with epidermal cells through pathways such as FGF, IGFBP, and BMP signaling. The BMP signaling pathway has been proven crucial in regulating the HF cycle and promoting follicular cell proliferation (Kulessa et al., 2000; Massague and Chen, 2000). This study suggests it may modulate HF activity through the coordinated action of ligands like BMP4, BMP2, and BMP7, which interact with receptors expressed across nearly all cell types. This broad regulatory capacity aligns with previous reports (Toledano et al., 2019). Similarly, we also found that the FGF signaling pathway involves coordinated action by multiple ligands, but its receptors have distinct roles: FGFR1 is almost exclusively localized to DPCs, consistent with earlier research (Kulessa et al., 2000), leading to the speculation that its primary function is autoregulation within DPCs. In contrast, FGFR2 is widely expressed in various cell types including DPCs, the IRS, and SG. We hypothesize that it primarily mediates downstream signal transduction, playing a role in HF growth and development, which is also supported by existing literature (Wang et al., 2025b; Keller et al., 2004). Additionally, we found that the PDGF, DHEA, and SLIT signaling pathways might also be influenced by DPCs. The PDGF pathway has been confirmed as indispensable for HF induction and formation (Rezza et al., 2015); DHEAS is a key factor influencing hair growth (Grymowicz et al., 2020); and an association exists between SLIT3 and FD (Yu et al., 2023). Therefore, we speculate that DPCs play a crucial role in regulating HF development.

Through reconstructing the differentiation process of the HF epidermal cell lineage, we found that HS and IRS are predominantly located in the later stages of the differentiation trajectory, while Mxs are concentrated in the early stages—consistent with previous literature reports (Schweizer et al., 2007). *FABP5* was identified among the differentially expressed genes. Prior studies indicate that this gene is highly expressed in differentiating HF cells (Charlotte and Watt, 2008), suggesting that during this period, cells are in a phase of vigorous metabolism and active biosynthesis. When reconstructing the differentiation trajectory of DPCs, we inferred that DPCs might exist in three functional states. KEGG analysis of the DP3 branch suggests this state may play a significant role in maintaining DPC identity and regulating HF growth and development (Xiaoxiang et al., 2022; Chang-min et al., 2015; Jiali et al., 2023). Furthermore, this branch highly expresses *POSTN*, which enhances the inductive capacity of DPCs by activating the focal adhesion signaling pathway (Gopee et al., 2024). The significant expression of the *COL1A1* gene in the DP1 branch, known to influence cell aggregation through adhesion and migration mechanisms (Cao and Feng, 2019; Wang et al., 2021), leads us to speculate that the functions of this branch are more closely related to the proliferative and differentiation potential of DPCs. Similarly, studies on the functional heterogeneity of DPCs in different states have long been conducted in mice. Research has shown that *SOX2* ^*-*^ positive and *SOX2* ^*-*^ negative DPCs exhibit functional divergence between two subpopulations. When *SOX2* ^*-*^ negative DPCs are absent, only zigzag HFs develop (Driskell et al., 2009). In our study, the classic marker gene *SOX2* was not screened out, which may indirectly support our findings: the lower the expression level of this gene, the better the development of zigzag HFs, thereby resulting in finer wool in fine-wool sheep. However, whether DPCs truly exist in three states to exert their physiological functions, as speculated in this study, requires further investigation and validation.

We also preliminarily explored the molecular mechanisms underlying FD. Prior to this study, several genes potentially affecting FD had been reported. For example, Zhao et al. (2021) identified *KIF16B*, UBE2E3, and *RHPN2* as associated with FD; Ma et al. (2023) performed single-cell RNA sequencing on skin tissues from Chinese Merino sheep (high follicular density) and Hotan sheep (low follicular density) and found that genes such as *COL1A1* and *LOC101116863* were correlated with FD in Chinese Merino sheep, while genes like *TNF* and *MAP2K2* were linked to follicular density in Hotan sheep. However, the specific regulatory mechanisms remain to be fully elucidated. In this study, by comparing gene expression across different cell types between the ultra-fine and fine wool groups, as well as between the medium-fine and fine wool groups, we identified DEGs in DPCs from the ultra-fine vs. fine comparison. Among these, *FABP4* (the fatty acid-binding protein) has been shown to exhibit distinct expression patterns in the skin of coarse-wool versus fine-wool sheep (Yongbin et al. 2016) and is involved in regulating wool rot resistance (Smith et al., 2010), relating to wool traits. *CRABP1* attracted particular attention due to its significant upregulation in DPCs. Previous research indicates that this gene plays an important role in cell growth by promoting retinoic acid transport and nuclear receptor interactions (Rendl et al., 2005), while also influencing HF size, shape, and growth cycle (Ma et al., 2019). Samples were collected in August when HFs are in an active growth phase. The marked increase in *CRABP1* expression suggests this gene may be associated with DPC proliferation. Early studies have shown that the number of DPCs determines hair thickness, shape, and the HF cycle; therefore, we speculate that *CRABP1* may play an important role in regulating FD. Additionally, we identified an interesting gene—*MYOC*. Previous research has suggested this gene may be associated with FD (Pu et al., 2024). In our study, *MYOC* was significantly downregulated in both comparison groups, leading us to hypothesize it may have a negative regulatory effect on FD. Therefore, future research could focus on the specific mechanisms of *MYOC* in DPC and HF development, and how it influences cellular behavior, thereby providing new insights and directions for a deeper investigation into HF biology and FD. The candidate DEGs identified in this study can serve as molecular markers for early breeding selection of ultra-fine wool sheep. By collecting skin tissue samples during the lamb stage and detecting the expression levels or functional variants of these genes using RT-qPCR or customized SNP chips, combined with a constructed gene signature-based scoring model, early prediction and selection for FD potential can be achieved. Furthermore, integrating these genes into a genomic selection framework will help shorten generation intervals and improve breeding efficiency, thereby upgrading traditional phenotypic selection to molecular-targeted precision breeding.

Based on the above analysis and combined with the cell proportion results across experimental groups, the ultra-fine group exhibited a higher proportion of DPCs. This suggests that DPCs may play a critical role in regulating FD. However, further research is required to thoroughly explore their regulatory mechanisms. Furthermore, the proportion of IMCs in the ultra-fine wool group was also lower, which is consistent with the findings of Jinshan et al. (2020), suggesting that the immune microenvironment may influence HF development at some stage, thereby indirectly regulating wool FD. When HFs are in a highly efficient developmental state, lower levels of IMCs may be sufficient to perform these signaling regulatory functions (Wei et al., 2025), the even lower proportion of IMCs in the ultra-fine wool group may reflect a more efficient and stable HF development microenvironment, with reduced dependence on immune signals.

Based on our research team’s established protocol for isolating, purifying, and characterizing DPCs (Song et al., 2024), we successfully obtained DPCs and performed cellular-level validation on the screened gene *CRABP1*. The proliferative capacity of DPCs is fundamental to maintaining HF population size and performing their “signaling hub” function, which is crucial for HF morphogenesis and cyclical regulation (Wang et al., 2025a). EDU and CCK-8 assay results indicated that the expression of *CRABP1* can promote the proliferation of DPCs. While exploring its effects on genes related to HF development, we found that overexpression of *CRABP1* upregulated the expression of *PCNA* and *CTNNB1*, while suppressing the expression of *SFRP2* and *BMP2*. *CTNNB1* is a downstream effector of the Wnt/β-catenin pathway (Watt et al., 2006). *PCNA* can regulate cellular proliferative capacity and cell cycle progression (Røst et al., 2022). *SFRP2* inhibits keratinocyte proliferation by suppressing Wnt activity (Bong-Kyu and Kim, 2014), thereby restricting HF growth. *BMP2* expression is nearly absent during the active growth phase but increases significantly during the resting phase (Sharov et al., 2003). Quantitative results indicate that this gene can influence DPC proliferation and also indirectly affect the HF microenvironment by regulating the Wnt/β-catenin and BMP signaling pathways, revealing its potential to modulate these pathways.

It should be noted that the use of half-sibling animals from the same father in this study unifies the genetic background to a certain extent, making variables such as follicular density and cell type differences more comparable across different FD groups. However, each group contains only two biological replicates (n = 2), which remains a limitation of this study. The small sample size may have constrained statistical power and could mean that the observed transcriptomic differences associated with FD are confounded by individual-specific effects or the particular genetic background of this lineage. Therefore, this research should be regarded as a preliminary exploratory study. Future validation in larger, more genetically diverse sheep populations, along with further *in vitro* and *in vivo* functional experiments, is needed to confirm the specific mechanisms of action of the identified candidate genes.

## 5 Conclusion

In summary, this study employed scRNA-seq to investigate the cellular heterogeneity within the skin tissues of fine-wool sheep. It identified 14 distinct cell types, delineated the cellular characteristics underlying HF development and differentiation, elucidated intercellular communication networks, and further revealed potential molecular mechanisms governing FD. These findings collectively provide novel insights for biological research on HF physiology and reproduction, as well as for breeding practices related to fine-wool sheep.

## Data Availability

The datasets generated and analyzed during the course of this study are not publicly available at this time, but may be obtained upon reasonable request to the corresponding author.

## Glossary

*APOD*: Apolipoprotein D
*BMP2*: Bone morphogenetic protein 2
*COL1AI*: Collagen type I alpha 1 chain
*CRABP1*: Cellular retinoic acid binding protein 1
*CTNNB1*: Catenin beta 1
CTS: Connective tissue sheath
*DCN*: Decorin
DEG: Differential gene expression
DPC: Dermal papilla cell
*FABP4*: Fatty acid binding protein 4
*FABP5*: Fatty acid binding protein 5
*FZD3*: Frizzled class receptor 3
*GATA3*: GATA binding protein 3
*GNAI3*: G protein subunit alpha i3
HE: Hematoxylin and eosin
HF: Hair follicle
HFSC: Hair follicle stem cell
*HOXC13*: Homeobox C13
HS: Hair shaft
*HSF5*: Heat shock transcription factor 5
IFE B: Interfollicular epidermis basal
IFE C: Interfollicular epidermis basal, cycling
IFE SB: Interfollicular epidermis super-basal
IRS: Inner root sheath
*ITGB6*: Integrin subunit beta 6
*KRT1*: Keratin 1
*KRT15*: Keratin 15
*KRT71*: Keratin 71
*KRTDAP*: Keratinocyte differentiation associated protein
*LEF1*: Lymphoid enhancer binding factor 1
*LGR5*: Leucine rich repeat containing G protein-coupled receptor 5
*LUM*: Lumican
Mx: Matrix
ORS: Outer root sheath
*PCNA*: Proliferating cell nuclear antigen
PF: Primary follicle
*PLCB2*: phospholipase C beta 2
*PRR9*: Proline rich 9
RT-qPCR: Real-time fluorescent quantitative PCR
scRNA-seq: Single-cell transcriptomics sequencing
SD: Secondary-derived follicle
SF: Secondary follicle
*SFRP2*: Secreted frizzled related protein 2
SG: Sebaceous gland
*SLIT3*: Slit guidance ligand 3
*SMAD3*: SMAD family member 3
SNP: Single nucleotide polymorphism
SO: Original secondary follicle
*SOX2*: SRY-box transcription factor 2
*SOX9*: SRY-box transcription factor 9
SW: Sweat gland
*TAGLN*: Transgelin
*TIMP1*: TIMP1 metallopeptidase inhibitor 1
*TOP2A*: DNA topoisomerase II alpha
UMAP: Uniform manifold approximation and projection
WFD: Wool fiber diameter
*ZNF280A*: Zinc finger protein 280A
α-SMA: Actin alpha 1
β-actin (ACTB): Actin beta

## References

[B1] Sharov S. A. Lorin W. Y S. T. Siebenhaar F. Atoyan R. Reginato A. M. (2003). Noggin overexpression inhibits eyelid opening by altering epidermal apoptosis and differentiation. EMBO Journal 22 (12), 2992–3003. 10.1093/emboj/cdg291 12805214 PMC162143

[B2] Bong-Kyu K. Kim Y. S. (2014). Expression of sfrp2 is increased in catagen of hair follicles and inhibits keratinocyte proliferation. Ann. Dermatology 26 (1), 79–87. 10.3342/anndermatol.2014.26.1.79 PMC3956799 24648690

[B3] Cao W. Feng Y. (2019). LncRNA XIST promotes extracellular matrix synthesis, proliferation and migration by targeting miR-29b-3p/COL1A1 in human skin fibroblasts after thermal injury. Biol. Res. 52, 52. 10.1186/s40659-019-0260-5 31540582 PMC6754631

[B4] Cédric B. Elaine F. (2009). Epidermal homeostasis: a balancing act of stem cells in the skin. Nat. Reviews. Mol. Cell Biology 10 (3), 207–217. 10.1038/nrm2636 PMC2760218 19209183

[B5] Chang J. Meng F. Zhang R. Feng J. Liu Y. Zhang J. (2024). Screening and expression validation of key proteins for secondary hair follicle growth in cashmere goats based on iTRAQ quantitative proteomics technology. Front. Veterinary Sci. 11, 1441074. 10.3389/fvets.2024.1441074 PMC11520223 39474271

[B6] Chang-min L. Yan-ping Y. Xian-cai C. Hai-hong L. Bo-zhi C. Yang L. (2015). Expression of Wnt/β-catenin signaling, stem-cell markers and proliferating cell markers in rat whisker hair follicles. J. Molecular Histology 46 (3), 233–240. 10.1007/s10735-015-9616-5 25832347

[B7] Charlotte A. Watt F. M. (2008). Dynamic regulation of retinoic acid-binding proteins in developing, adult and neoplastic skin reveals roles for β-catenin and notch signalling. Dev. Biol. 324 (1), 55–67. 10.1016/j.ydbio.2008.08.034 18805411

[B8] Chovatiya G. Ghuwalewala S. Walter L. D. Cosgrove B. D. Tumbar T. (2021). High‐resolution single‐cell transcriptomics reveals heterogeneity of self‐renewing hair follicle stem cells. Exp. Dermatol. 30 (4), 457–471. 10.1111/exd.14262 33319418 PMC8016723

[B9] Chu X. Zhou Z. Qian X. Shen H. Cheng H. Zhang J. (2025). Functional regeneration strategies of hair follicles: advances and challenges. Stem Cell Res. and Ther. 16 (1), 1–12. 10.1186/s13287-025-04210-y 39985119 PMC11846195

[B10] Chunyan M. Ben J. Pascal S. Paul A. O. Denis J. H. (2006). Generation of the primary hair follicle pattern. Proc. Natl. Acad. Sci. U. S. A. 103 (24), 9075–9080. 10.1073/pnas.0600825103 16769906 PMC1482568

[B45] Driskell R. R. Adam G. B K. J. Mulder K. W. Watt F. M. (2009). Sox2-positive dermal papilla cells specify hair follicle type in mammalian epidermis. Development 136, 2815–2823. 10.1242/dev.038620 19605494 PMC2730408

[B11] Elliott K. Stephenson T. J. Messenger A. G. (1999). Differences in hair follicle dermal papilla volume are due to extracellular matrix volume and cell number: implications for the control of hair follicle size and androgen responses. J. Investigative Dermatology 113 (6), 873–877. 10.1046/j.1523-1747.1999.00797.x 10594724

[B12] Feng Y. Rui L. Cun Z. Tianyu C. Juntao G. Yuchun X. (2022). Single-cell sequencing reveals the new existence form of dermal papilla cells in the hair follicle regeneration of cashmere goats. Genomics 114 (2), 110316. 10.1016/j.ygeno.2022.110316 35202721

[B13] Ge W. Zhang W. D. Zhang Y. L. Zheng Y. J. Li F. Wang S. H. (2022). A single-cell transcriptome atlas of Cashmere goat hair follicle morphogenesis. 10.1016/j.gpb.2021.07.003 PMC8864196 34534715

[B14] Gerhards N. M. Sayar B. S. Origgi F. C. Galichet A. Müller E. J. Welle M. M. (2016). Stem cell-associated marker expression in CanineHair follicles. J. Histochem. and Cytochem. 64 (3), 190–204. 10.1369/0022155415627679 26739040 PMC4810799

[B15] Gopee N. H. Winheim E. Olabi B. Admane C. Foster A. R. Huang N. (2024). A prenatal skin atlas reveals immune regulation of human skin morphogenesis. Nature 635 (8039), 679–689. 10.1038/s41586-024-08002-x 39415002 PMC11578897

[B16] Grymowicz M. Rudnicka E. Podfigurna A. Napierala P. Smolarczyk R. Smolarczyk K. (2020). Hormonal effects on hair follicles. Int. J. Mol. Sci. 21 (15), 5342. 10.3390/ijms21155342 32731328 PMC7432488

[B17] Gu L. H. Coulombe P. A. (2007). Keratin function in skin epithelia: a broadening palette with surprising shades. Curr. Opin. Cell Biol. 19, 13–23. 10.1016/j.ceb.2006.12.007 17178453

[B18] Gupta K. Levinsohn J. Linderman G. Chen D. Sun T. Y. Dong D. (2019). Single-cell analysis reveals a hair follicle dermal niche molecular differentiation trajectory that begins prior to morphogenesis. Dev. Cell 48 (1), 17–31. 10.1016/j.devcel.2018.11.032 30595533 PMC6361530

[B19] Haig A. Connie C. Vivienne C. H. Mudit G. Manvendra K. M. Jonathan A. E. (2014). Semaphorin 3d and semaphorin 3e direct endothelial motility through distinct molecular signaling pathways. J. Biological Chemistry 289 (26), 17971–17979. 10.1074/jbc.M113.537977 PMC4140303 24825896

[B20] He W. Ye J. Xu H. Lin Y. Zheng Y. (2020). Differential expression of α6 and β1 integrins revealsepidermal heterogeneity at single‐cell resolution. J. Cell. Biochem. 121 (3), 2664–2676. 10.1002/jcb.29487 31680320

[B21] Huang D. Ding H. Wang Y. Cheng G. Wang X. Leng T. (2023). Hair follicle transcriptome analysis reveals differentially expressed genes that regulate wool fiber diameter in angora rabbits. Biology 12 (3), 445. 10.3390/biology12030445 36979137 PMC10045444

[B22] Huang D. Ding H. Wang Y. Wang X. Zhao H. (2024). Integration analysis of hair follicle transcriptome and proteome reveals the mechanisms regulating wool fiber diameter in angora rabbits. Int. J. Mol. Sci. 25 (6), 15. 10.3390/ijms25063260 38542234 PMC10970426

[B23] Jaks V. Barker N. Kasper M. van Es J. H. Snippert H. J. Clevers H. (2008). Lgr5 marks cycling, yet long-lived, hair follicle stem cells. Nat. Genet. 40 (11), 1291–1299. 10.1038/ng.239 18849992

[B24] Ji S. F. Zhu Z. Y. Sun X. Y. Fu X. B. (2021). Functional hair follicle regeneration: an updated review. Signal Transduct. Target. Ther. 6 (1), 66. 10.1038/s41392-020-00441-y 33594043 PMC7886855

[B25] Jiali L. Bohao Z. Shuyu Y. Dai Y. Zhang X. Yang N. (2023). Dermal papilla cell-derived exosomes regulate hair follicle stem cell proliferation *via* LEF1. Int. J. Mol. Sci. 24 (4), 3961. 10.3390/ijms24043961 36835374 PMC9964005

[B26] Jinshan Z. Huaiyuan Q. Jingjing X. Nan L. Rongwei H. PerezCampo F. M. (2020). Discovery of genes and proteins possibly regulating mean wool fibre diameter using cDNA microarray and proteomic approaches. Sci. Reports 10 (1), 7726. 10.1038/s41598-020-64903-7 PMC7206055 32382132

[B27] Joost S. Annusver K. Jacob T. Sun X. Dalessandri T. Sivan U. (2020). The molecular anatomy of mouse skin duringhair growth and rest. Cell Stem Cell 26 (3), 441–457. 10.1016/j.stem.2020.01.012 32109378

[B28] Keller U. A. D. Krampert M. Kümin A. Braun S. Werner S. (2004). Keratinocyte growth factor:effects on keratinocytes and mechanisms of action. Eur. J. Cell Biol. 83 (11-12), 607–612. 10.1078/0171-9335-00389 15679105

[B29] Kerstin F. Tanja S. Motonobu N. Ursula H. Ralf P. (2005). Towards dissecting the pathogenesis of retinoid-induced hair loss: all-trans retinoic acid induces premature hair follicle regression (catagen) by upregulation of transforming growth factor-beta2 in the dermal papilla. J. Investigative Dermatology 124 (6), 1119–1126. 10.1111/j.0022-202X.2005.23686.x 15955085

[B30] Kulessa H. Tirk G. Hogan B. L. (2000). Inhibition of Bmp signaling affects growth and differentiation in the anagen hair follicle. EMBO J. 19 (24), 6664–6674. 10.1093/emboj/19.24.6664 11118201 PMC305899

[B31] Li C. He X. Wu Y. Li J. Zhang R. An X. (2024). Single-cell transcriptome sequence profiling on the morphogenesis of secondary hair follicles in ordos fine-wool sheep. Int. J. Mol. Sci. 25 (1), 584. 10.3390/ijms25010584 38203755 PMC10779399

[B33] Ma H. Y. (2024). Identification of genes associated with wool density traits. Xinjiang Agricultural University.

[B49] Ma S. Li L. Xixia H. (2023). Transcriptome analysis reveals genes associated with wool fineness in merinos. Peer J. 11, e15327. 10.7717/peerj.15327 37250719 PMC10215774

[B34] Ma S. Zhou G. Chen Y. (2018). Effects of all-trans retinoic acid on goat dermal papilla cells cultured *in vitro* . Electron. J. Biotechnol. 34, 3443–5340. 10.1016/j.ejbt.2018.05.004

[B35] Ma S. Wang Y. Zhou G. Ding Y. Yang Y. Wang X. (2019). Synchronous profiling and analysis of mRNAs and ncRNAs in the dermal papilla cells from cashmere goats. BMC Genom 20, 512. 10.1186/s12864-019-5861-4 31221080 PMC6587304

[B36] Marcin M. Gerlind H. Til A. Rudolf E. L. Reinhard W. (2013). Measuring the regulation of keratin filament network dynamics. Proc. Natl. Acad. Sci. U. S. A. 110 (26), 10664–10669. 10.1073/pnas.1306020110 23757496 PMC3696749

[B37] Massague J. Chen Y. G. (2000). Controlling TGF-beta signaling. Genes Dev. 14 (6), 627. 10.1101/gad.14.6.627 10733523

[B38] Moore G. P. M. Jackson N. Lax J. (1989). Evidence of a unique developmental mechanism specifying both wool follicle density and fibre size in sheep selected for single skin and fleece characters. Genet. Res. 53 (1), 57–62. 10.1017/s0016672300027865 2714646

[B39] Okano J. Levy C. Lichti U. Sun H. W. Yuspa S. H. Sakai Y. (2012). Cutaneous retinoic acid levels determine hair follicle development and downgrowth. J. Biol. Chem. 287 (47), 39304–39315. 10.1074/jbc.M112.397273 23007396 PMC3501026

[B40] Oshima H. Rochat A. Kedzia C. Kobayashi K. Barrandon Y. (2001). Morphogenesis and renewal of hair follicles from adult multipotent stem cells. Cell 104 (2), 233–245. 10.1016/s0092-8674(01)00208-2 11207364

[B41] Paus R. Handjiski B. Czarnetzki B. M. Eichmüller S. (1994). Biology of the hair follicle. Der Hautarzt 45 (11), 808–825. 10.1007/s001050050180 7822211

[B42] Polkoff K. M. Gupta N. K. Green A. J. Murphy Y. Chung J. Gleason K. L. (2022). LGR5 is a conserved marker of hair follicle stem cells in multiple species and is present early and throughout follicle morphogenesis. Sci. Reports 12 (1), 9104. 10.1038/s41598-022-13056-w 35650234 PMC9160037

[B43] Pu X. Ma S. Zhao B. Tang S. Lu Q. Liu W. (2024). Transcriptome meta-analysis reveals the hair genetic rules in six animal breeds and genes associated with wool fineness. Front. Genet. 14 (1), 1401369. 10.3389/fgene.2024.1401369 38948362 PMC11211574

[B44] Qingbo Z. Na Y. Pengjia B. Xiaolan Z. Fubin W. Lanhua M. (2023). Correction: construction of transcriptome atlas of white yak hair follicle during anagen and catagen using single-cell RNA sequencing. BMC Genomics 24 (1), 60. 10.1186/s12864-022-09059-y 36732700 PMC9896799

[B46] Rendl M. Lewis L. Fuchs E. (2005). Molecular dissection of mesenchymal–epithelial interactions in the hair follicle. PLoS Biol. 3 (11), e331. 10.1371/journal.pbio.0030331 16162033 PMC1216328

[B47] Rezza A. Sennett R. Tanguy M. Clavel C. Rendl M. (2015). Pdgf signalling in the dermis and in dermal condensates is dispensable for hair follicle induction and formation. Exp. Dermatol. 24 (6), 468–470. 10.1111/exd.12672 25708924 PMC4943754

[B32] Røst L. M. Ræder S. B. Olaisen C. Søgaard C. K. Sharma A. Bruheim P. (2022). PCNA regulates primary metabolism by scaffolding metabolic enzymes. Oncogene 42 (8), 613–624. 10.1038/s41388-022-02579-1 36564470 PMC9937922

[B48] Schweizer J. Langbein L. Rogers M. A. Winter H. (2007). Hair follicle-specific keratins and their diseases. Exp. Cell Res. 313 (10), 2010–2020. 10.1016/j.yexcr.2007.02.032 17428470

[B50] Shanhe W. Tianyi W. Jingyi S. Yue L. Y. Zehu Y. Wei S. (2021). Single-cell transcriptomics reveals the molecular anatomy of sheep hair follicle heterogeneity and wool curvature. Front. Cell Dev. Biol. 9, 800157. 10.3389/fcell.2021.800157 34993204 PMC8724054

[B51] Smith W. J. Li Y. Ingham A. Collis E. McWilliam S. M. Dixon T. J. (2010). A genomics-informed, SNP association study reveals FBLN1 and FABP4 as contributing to resistance to fleece rot in Australian Merino sheep. Bmc Veterinary Res. 6 (1), 27. 10.1186/1746-6148-6-27 20500888 PMC2886023

[B52] Song Y. Li Y. Lu Z. Yue L. Xiao T. Yang B. (2024). FGF20 secreted from dermal papilla cells regulate the proliferation and differentiation of hair follicle stem cells in fine-wool sheep. J. Animal Physiology Animal Nutrition 109 (3), 655–666. 10.1111/jpn.14081 39704013 PMC12091089

[B53] Sun W. (2024). CRABP1 enhances the proliferation of the dermal papilla cells of Hu sheep through the Wnt/β-catenin pathway. Genes 15 (10), 1291–1291. 10.3390/genes15101291 39457415 PMC11507202

[B54] Tina J. Karl A. Paulo C. Tim D. Christina K. Levron C. L. (2023). Molecular and spatial landmarks of early mouse skin development. Dev. Cell 58 (20), 2140–2162.e5. 10.1016/j.devcel.2023.07.015 37591247 PMC11088744

[B55] Toledano S. Nir-Zvi I. Engelman R. Kessler O. Neufeld G. (2019). Class-3 semaphorins and their receptors: potent multifunctional modulators of tumor progression. Int. J. Mol. Sci. 20 (3), 556. 10.3390/ijms20030556 30696103 PMC6387194

[B56] Wang M. M. (2023). The effect of Thsd4 on promoting hair follicle. Chongqingh University.

[B57] Wang J. Sui J. Mao C. Li X. R. Chen X. Y. Liang C. C. (2021). Identification of key pathways and genes related to the development of hair follicle cycle in cashmere goats. Genes (2). 12 (2), 180. 10.1093/BURNST/TKAC022 PMC7911279 33513983

[B58] Wang Y. Jiang Y. Ni G. Li S. Balderson B. Zou Q. (2024). Integrating single‐cell and spatial transcriptomics reveals heterogeneity of early pig skin development and a subpopulation with hair placode formation. Adv. Sci. 11 (20), 2306703. 10.1002/advs.202306703 PMC11132071 38561967

[B59] Wang M. Wang M. Jiang J. Li K. Liang H. Wang N. O. (2025a). THSD4 promotes hair growth by facilitating dermal papilla and hair matrix interactions. Theranostics 15 (8), 3571–3588. 10.7150/thno.103221 40093891 PMC11905124

[B60] Wang N. Zhang W. Zhong Z. Zhou X. Shi X. Wang X. (2025b). FGF7 secreted from dermal papillae cell regulates the proliferation and differentiation of hair follicle stem cell. J. Integr. Agric. 24 (09), 3583–3597. 10.1016/j.jia.2023.10.012

[B61] Watt F. M. Lo C. C. Silva-Vargas V. (2006). Epidermal stem cells: an update. Curr. Opin. Genet. and Dev. 16 (5), 518–524. 10.1016/j.gde.2006.08.006 16919447

[B62] Wei Y. Gu J. Zhao Z. Gu B. (2025). Immunological control of skin development: from homeostasis to developmental pathologies. Front. Immunology 16, 1685633. 10.3389/fimmu.2025.1685633 41376622 PMC12685724

[B63] Xiao-Jing Z. YuDong L. Zhong-Min D. Xiaoyun Z. XueQin Y. Yan L. (2014). BMP-FGF signaling axis mediates Wnt-induced epidermal stratification in developing mammalian skin. PLoS Genetics 10 (10), e1004687. 10.13881/j.cnki.hljxmsy.2017.2159 25329657 PMC4199507

[B64] Xiaoxiang W. Yinghui L. Jia H. Jingru W. Xiaodong C. Ronghua Y. (2022). Regulation of signaling pathways in hair follicle. Stem Cells 10 tkac022. 10.1093/burnst/tkac022 PMC9250793 35795256

[B65] Xuxu H. (2023). Dissecting the transcriptional atlas and molecular characteristics of embryonic hair follicles of Shanbei White Cashmere goat at single-cellre solution. Northwest A&F University.

[B66] Ying S. Shiraishi A. Kao C. W. Converse R. L. Funderburgh J. L. Swiergiel J. (1997). Characterization and expression of the mouse lumican gene. J. Biol. Chem. 272, 30306–30313. 10.1074/jbc.272.48.30306 9374517

[B67] Yongbin L. Yunxia Q. Shaoyin F. Xiaolong H. Wengguang Z. Weiheng R. (2016). Transcriptome analysis reveals skin lipid metabolism related to wool diameter in sheep. 317–318.

[B68] Yue L. Lu Z. Guo T. Liu J. Yuan C. Yang B. (2023). Association of SLIT3 and ZNF280B gene polymorphisms with wool fiber diameter. Animals 13 (22), 10. 10.3390/ani13223552 38003169 PMC10668676

[B69] Yue L. Lu Z. Guo T. Liu J. Yang B. Yuan C. (2024). Key genes and metabolites that regulate wool fibre diameter identified by combined transcriptome and metabolome analysis. Genomics 116 (5), 11. 10.1016/j.ygeno.2024.110886 38880312

[B70] Zeng X. C. Yu Y. S. Cao Y. Jin G. H. (2017). Application progress of FGF5 in research on hair follicle development of cashmere goats. Heilongjiang Animal Husb. Veterinary Med. 23 (23), 82–85+294.

[B71] Zhang B. Chen T. (2024). Local and systemic mechanisms that control the hair follicle stem cell niche. Nat. Rev. Mol. Cell Biol. 25 (2), 14–100. 10.1038/s41580-023-00662-3 37903969

[B72] Zhang W. Jin M. Li T. Lu Z. Wang H. Yuan Z. (2023). Whole-genome resequencing reveals selection signal related to sheep wool fineness. Animals 13 (18), 2944. 10.3390/ani13182944 37760343 PMC10526036

[B73] Zhang J. P. Xiao M. Fang J. B. Huang D. L. Zhao Y. J. (2025). Phenotypic, transcriptomic, and genomic analyses reveal the spatiotemporal patterns and associated genes of coarse hair density in goats. Zoological Res. 46 (4), 825–840. 10.24272/j.issn.2095-8137.2025.034 40640980 PMC12464370

[B74] Zhao H. Guo T. Lu Z. Liu J. Zhu S. Qiao G. (2021). Genome-wide association studies detects candidate genes for wool traits by re-sequencing in Chinese fine-wool sheep. BMC Genomics 22 (1), 127. 10.1186/s12864-021-07399-3 33602144 PMC7893944

