# Supplementary material for: Deciphering the cellular landscape and genetic underpinnings of fiber diameter determined by dermal papilla cells in fine-wool sheep
Source: Front Cell Dev Biol. 2026 Jun 1;14:1764812. doi: 10.3389/fcell.2026.1764812 (PMC13265513; doi:10.3389/fcell.2026.1764812)
Supplement: Supplementary file 1 [file Supplementaryfile1.docx]

**Supplementary Information**

**Table S1**. siRNA Sequence Information

| **Gene name** | **Sequence (5’-3’)** |
| --- | --- |
| siRNA*-CRABP1*-121 | AUGCGCAGCAGCGAGAAUUTT |
|  | AAUUCUCGCUGCUGCGCAUTT |
| siRNA*-CRABP1*-483 | CACGAGGAUUUAUGUUCGGTT |
|  | CCGAACAUAAAUCCUCGUGTT |
| siRNA*-CRABP1*-190 | GCCGUGGCGGCUGCGUCCATT |
|  | UGGACGCAGCCGCCACGGCTT |
| siRNA*-CRABP1*-312 | GGAGGAGACCGUGGACGGATT |
|  | UCCGUCCACGGUCUCCUCCTT |
| siRNA-NC | UUCUCCGAACGUGUCACGUTT |
|  | ACGUGACACGUUCGGAGAATT |

**Table S2**. RT-qPCR Primers

| **Gene name** | **Forward primer sequence** | **Reverse primer sequence** |
| --- | --- | --- |
| *SFRP2* | GACAACGACCTTTGCATCCC | ATACCTTCGGAGCTTCCTCG |
| *CTNNB1* | AAGACATCACTGAGCCTGCC | GTCCGTAGTGAAGGCGAACA |
| *BMP2* | CACACCCTACCCGAGATTGG | CTGAGTCCCCAGTAATCCGC |
| *PCNA* | CGTGAACCTCACCAGCATGTC | GTGTCCGCATTATCTTCAGCC |
| *β-actin* | CAGTCGGTTGGATGGAGCAT | AGGCAGGGACTTCCTGTAAC |
| *CRABP1* | GGTGCGGAAGTAAGCTTGGA | CGTCGAAATTCTCGCTGCTG |

**Table S3.** Antibody information

| Antibody | Source | Catalog Number |
| --- | --- | --- |
| KRT15 | Boster | A-06791-4 |
| KRT35 | Bioss | bs-16826R |
| KRT71 | Bioss | bs-16830R |
| VIM | Boster | PB9359 |
| HOXC13 | Bioss | bs-13599R |
| TAGLN | Boster | A03962-2 |
| Goat anti-rabbit IgG | Servicebio | GB21303 |

**Table S4.** Information on the FD, LID, and count of wool FD from the experimental sheep in this study

| Number | LID | Sire LID | WFD | Shepherd | Wool count |
| --- | --- | --- | --- | --- | --- |
| 1 | 230147 | 211200 | 16.3 | Huping | 80 |
| 2 | 230264 | 211200 | 16.8 | Huping |  |
| 3 | 230190 | 211200 | 19.6 | Huping | 70 |
| 4 | 230184 | 211200 | 19.6 | Huping |  |
| 5 | 230171 | 211200 | 20.2 | Huping | 66 |
| 6 | 230186 | 211200 | 21.4 | Huping |  |

**Table S5.** Comparison of the number of genes detected and the number of UMIs detected in each cell

| Sample | CX1 | CX2 | X1 | X2 | ZX1 | ZX2 |
| --- | --- | --- | --- | --- | --- | --- |
| Estimated number of cells | 8,184 | 10,580 | 11,230 | 10,442 | 9,312 | 9,984 |
| Cell number filtered | 7655 | 9746 | 10262 | 9603 | 8597 | 9185 |
| Percentage of valid barcodes | 97.00% | 96.90% | 96.80% | 96.60% | 96.60% | 95.80% |
| Mean reads per cell | 40,670 | 30,885 | 30,400 | 31,603 | 35,862 | 35,063 |
| Median genes per cell | 1,432 | 1,694 | 1,809 | 1,922 | 1,896 | 1,965 |
| Total genes detected | 18,891 | 19,469 | 19,708 | 19,516 | 19,565 | 19,783 |
| Reads mapped to genome | 96.30% | 96.40% | 96.10% | 96.30% | 96.30% | 96.20% |

**
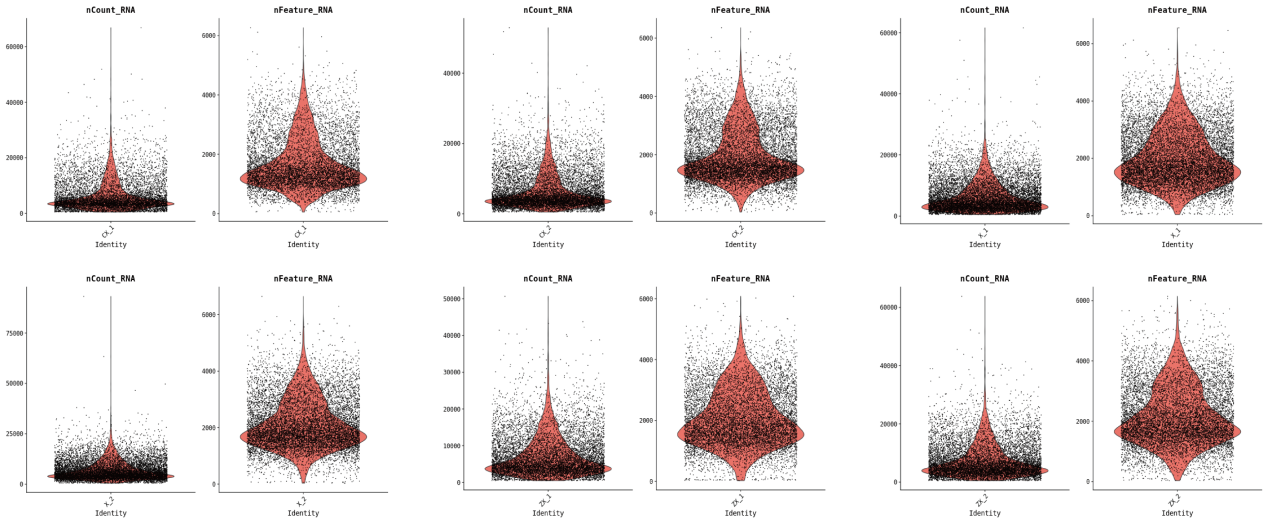
**

**Fig. S1** Detailed quality matrices for all datasets in this study revealed by CellRanger.

**
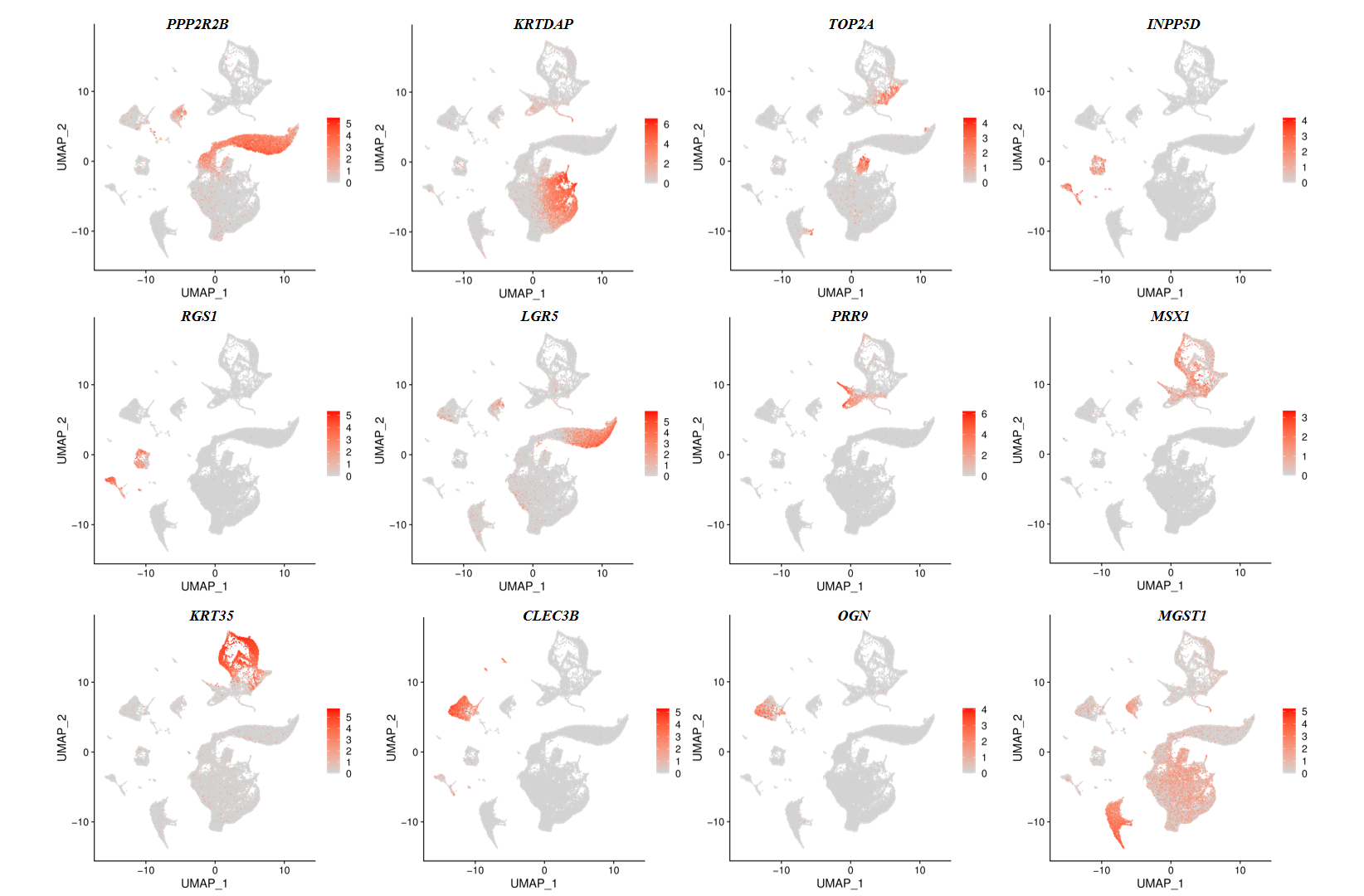
**

**Fig. S2** Evaluating key cell type markers across all single cells in the UMAP plot.

**Table S6:** Cell types and their corresponding marker genes analyzed from scRNA-seq.

| **Number** | **Cell type** | **Markers** |
| --- | --- | --- |
| 1 | Interfollicular epidermis basal (IFE B) | *POSTN*, *KRT15* |
| 2 | Interfollicular epidermis super-basal (IFE SB) | *KRTDAP*, *KRT1*, *KRT15*, *SBSN* |
| 3 | Interfollicular epidermis basal, cycling (IFE C) | *TOP2A*, *BIRC5*, *KRT15* |
| 4 | Dermal papilla (DP) | *LUM*, *APOD*, *CLEC3B* |
| 5 | Matrix (Mx) | *MSX1*, *HOXC13*, *UBE2C* |
| 6 | Inner root sheath (IRS) | *PRR9*, *KRT71*, *GATA3* |
| 7 | Hair shaft (HS) | *KRT35*, *LEF1* |
| 8 | Immune cells (IMC) | *SRGN*, *INPP5D*, *CD3E*, *RGS1* |
| 9 | Sebaceous gland (SG) | *SCD*, *MGST1* |
| 10 | Hair follicle stem cells (HFSC) | *LHX2*, *CPXM2* |
| 11 | Endothelial cells (EC) | *PLVAP*, *VWF*, *KDR* |
| 12 | Pricytes | *ABCC9*, *TPM2*, *EBF2* |
| 13 | Outer root sheath (ORS) | *SFRP1*, *PPP2R2B*, *TAGLN*, *LGR5* |
| 14 | Unknown | -- |

**
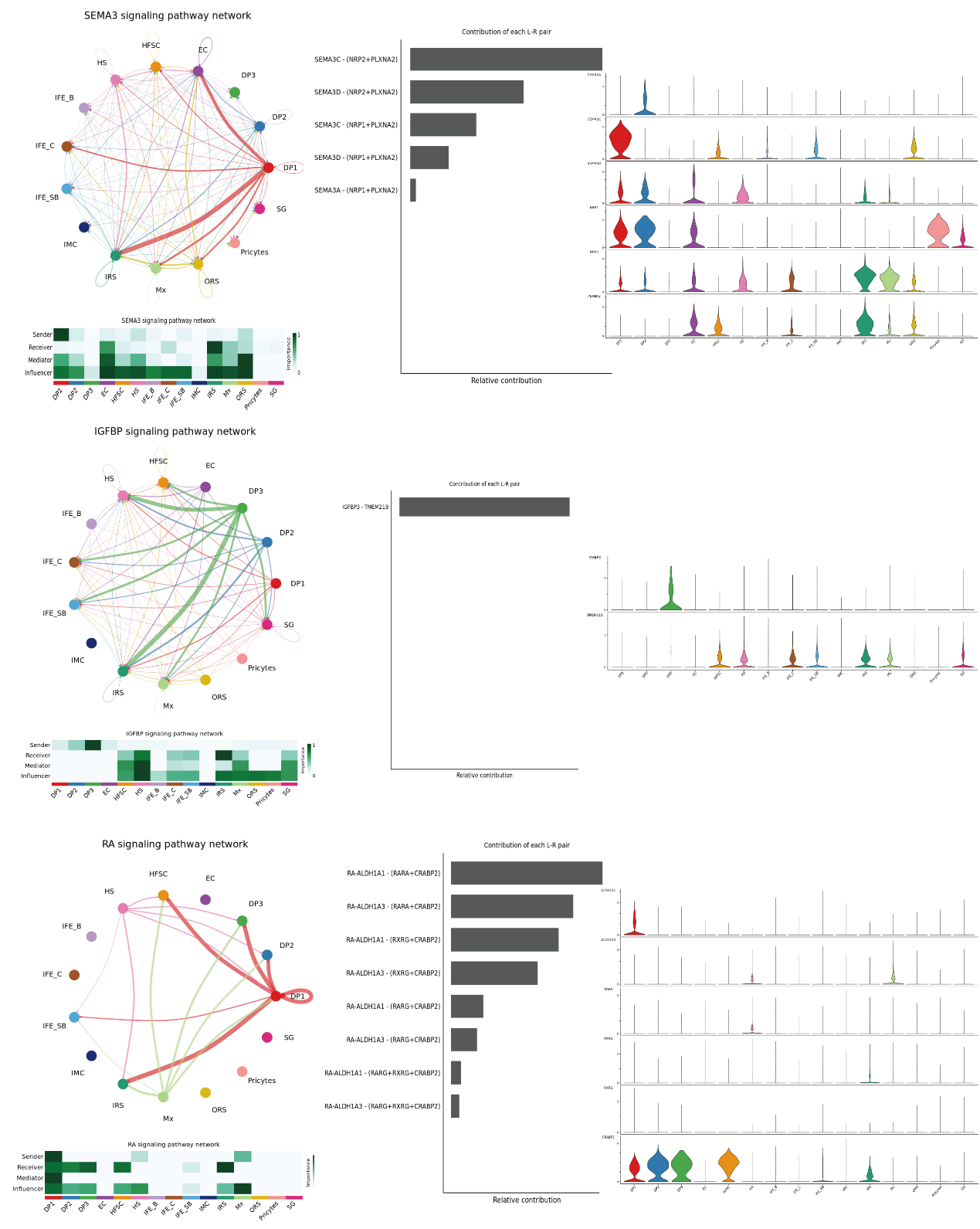
**

**Fig. S3** Violin plots displaying the output intensity, key ligand-receptor pairs, and related gene expression for the SEMA3, IGFBP and RA signaling pathway across multiple cell types.

**
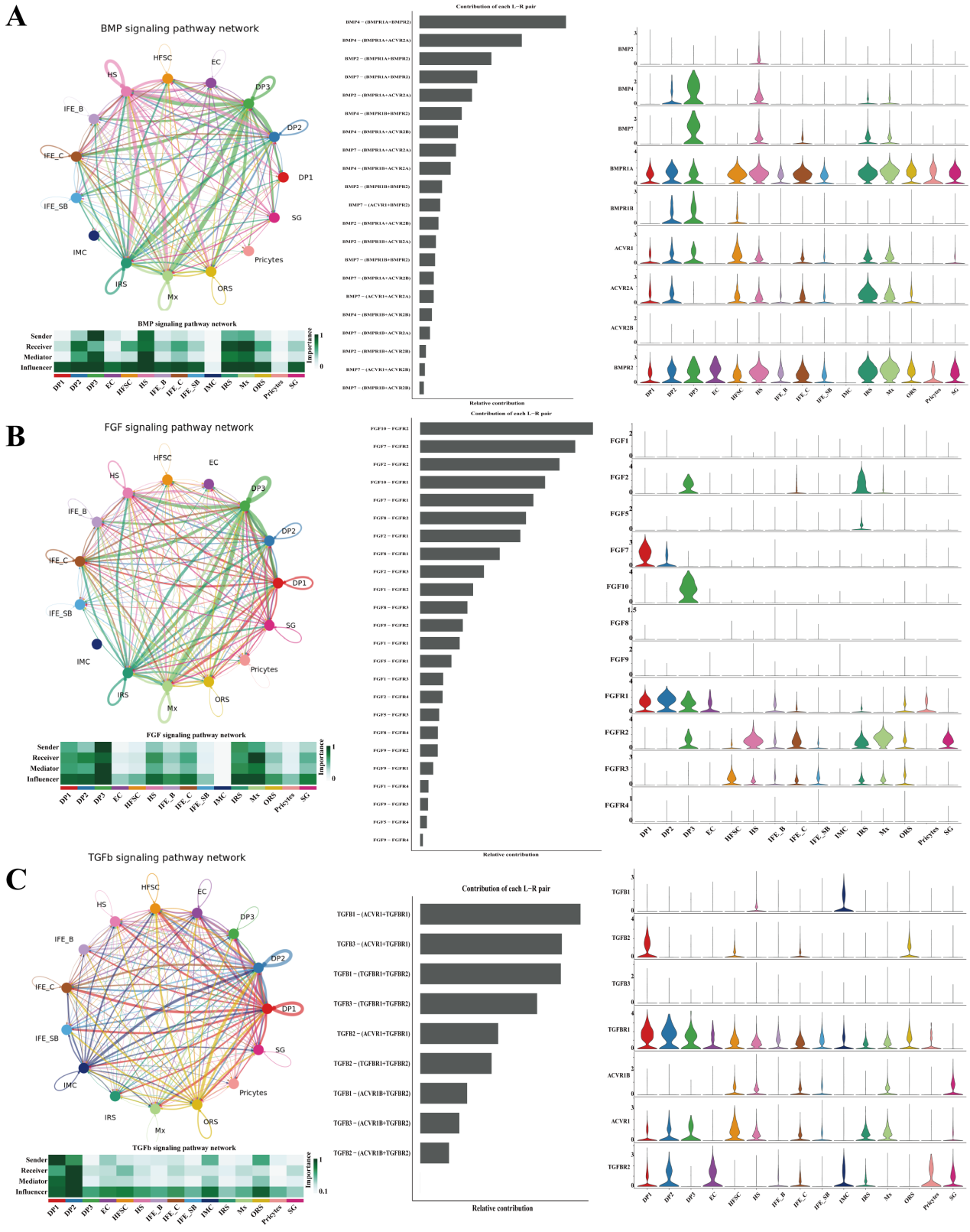
**

**Fig S4.** BMP, FGF, and TGF-β Signaling Pathway Networks

**A:** Violin plots depicting the output intensity, key ligand-receptor pairs, and related gene expression for the BMP signaling pathway across different cell types. **B**: Violin plots illustrating the output intensity, key ligand-receptor pairs, and related gene expression for the FGF signaling pathway across various cell types. **C**: Violin plots displaying the output intensity, key ligand-receptor pairs, and related gene expression for the TGF-β signaling pathway across multiple cell types.

**
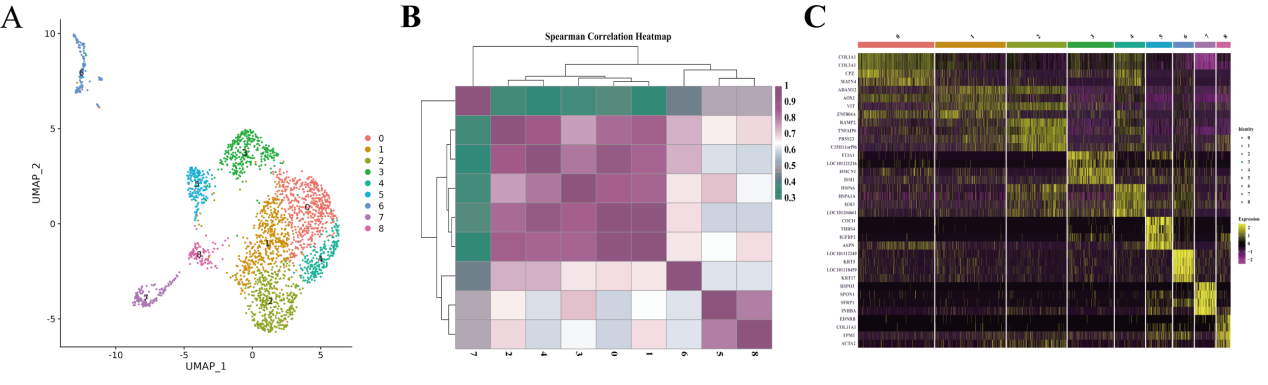
**

**Fig S5.** Subpopulations of DPCs and Gene Expression Heat Map

**A**: After re-grouping the DPCs, eight clusters were obtained; **B**: Heatmap of correlation between clusters in DPCs subpopulations; **C**: DEGs map of individual cell clusters in DPCs subpopulations;
